# Editorial: Mpox: understanding the scientific gaps to combat the threat

**DOI:** 10.3389/fcimb.2024.1538718

**Published:** 2025-01-06

**Authors:** Rehab M. Elsaid Tash, Mohd Ahmar Rauf, Rehab Hosny El-sokkary

**Affiliations:** ^1^ Medical Microbiology and Immunology Department, Faculty of Medicine, Zagazig University, Zagazig, Egypt; ^2^ Barbara Ann Karmanos Cancer Institute, School of Medicine, Wayne State University, Detroit, MI, United States

**Keywords:** mpox, knowledge, coinfection, one health, STDs

Mpox (formerly Monkeypox) is a viral zoonotic infectious disease caused by an Orthopoxvirus first detected in humans in 1970, and historically confined to West and Central Africa. By August 2024, the World Health Organization declared mpox a public health emergency of international concern (PHEIC). This PHEIC determination is the second in two years relating to mpox. The emergence of a new clade of mpox (clade Ib), its rapid spread in the Democratic Republic of the Congo mainly through sexual networks, and the reporting of cases in several neighboring countries are factors that raised concerns about the possibility of an upcoming global outbreak (World Health Organization, 2024). In parallel, Africa CDC declares mpox a Public Health Emergency of Continental Security (PHECS) (Africa Centres for Disease Control and Prevention, 2024).

The first PHEIC was July 23rd, 2022, when mpox outbreak in non-endemic countries (clade II) was reported. By May 2023, it was declared over after a sustained decline in global cases (World Health Organization, 2024).

The recent increase in mpox cases has led to critical inquiries regarding the virus’s etiology, evolution, and transmission dynamics. The global dissemination of mpox virus (MPXV) necessitates thoroughly re-evaluating its effects beyond endemic regions. It is critical to fill the current scientific gap to identify at-risk populations and design evidence-based preventive measures to control the current outbreak. Misdiagnosis and underdiagnosis of mpox can lead to larger and longer outbreaks, so clinical and laboratory diagnostic items should be updated regularly. Hence, the Research Topic “*Mpox: Understanding the scientific gaps to combat the threat*” aims to make mpox related dynamic changes readily available to health care practitioners, as well as the relevant worldwide community scope and information for writers.

In the current Research Topic, we got contributions from Africa and Asia. The topic encompasses a review of MPXV characteristics, and a perspective about the role of one health to prevent the re-emergence of the virus. In addition to, two research articles investigated knowledge, attitude, and perception of health professionals, and medical students about MPX and the willing of men having sex with men (MSM) to be vaccinated. Lastly, two articles discussed the clinical presentations and coinfection with other viral infections.

The study of Alakunle et al. presented a comprehensive review of the properties of MPXV by examining the virus’s ecology, genetics, and host interactions. Moreover, they presented its evolution, particularly clade IIb associated with the 2022 outbreaks, with an emphasis about the necessity for enhanced comprehension of host immune responses and viral modifications to effectively anticipate and mitigate future outbreaks. The resurgence of mpox was investigated in Nigeria by the study of (Ogunleye et al.). They highlighted the complex interaction of conditions that have facilitated its dissemination. Their analysis identified human activities, ecological disturbances, and biosecurity failures as the key factors in transmission. The cessation of smallpox vaccination, which previously offered cross-protection, increased susceptibility within the population. They underscored proactive surveillance, vaccination initiatives, and public health education as essential strategies to mitigate future outbreaks. Both articles advocate a collaborative One Health approach, highlighting the interrelation between human, animal, and environmental health in the management and prevention of mpox epidemics.

Frome Africa, the study of Amer et al. advocates the need for focused education to improve mpox prevention and control. In their survey of 1,034 Egyptian medical students and healthcare professionals, they reported that 55.3% had adequate knowledge about mpox whereas 44.5% and 39.8%, respectively, had positive attitudes and perception. Doctors, married people, and those over 40 showed a higher knowledge level. Males, urban residents, and nurses were associated with positive attitude, whereas married individuals, physicians, and pharmacy/laboratory staff were more likely to have positive perception.

The eastern hemisphere had its contribution tackling mpox in the Asian population. The study of Jia et al. investigated twenty Chinese cisgender males with confirmed mpox;95% of them reported having unprotected sex with men. STIs are present in 15 cases (75%); 13 (65%) were HIV-positive, ten (50%) had syphilis, and eight (40%) had HIV-syphilis co-infection. The majority of whom had well-controlled illnesses. Skin and anal lesions had the highest MPXV loads, indicating close contact transmission. Fever (85%), lymphadenopathy (55%), and skin lesions (85% anogenital) were common symptoms. Recovery took roughly 14 days, and the median incubation period was 8 days. Given the high prevalence of HIV and STI co-infection, the study places a strong emphasis on prevention for young gay males.

The readiness to vaccination was examined in the study of (Huang et al.). They surveyed 1,903 MSM in China to assess knowledge of mpox, concerns, and willingness to vaccinate. They stated that 94.1% of respondents supported vaccine promotion and 69.9% were aware of MPX. The majority of individuals (91.4%) agreed to get vaccinated against mpox. Those who prioritized MSM immunization, thought the vaccine was safe, supported government promotion of the vaccine, or had vaccinated friends were more willing. Married people and those who engage in frequent anal intercourse showed hesitancy. The researchers concluded that although there is a high level of vaccination willingness, MSM uptake can be improved by addressing safety concerns and utilizing social influence.

The clinical case report of Balingit et al. from the Philippines underscores the complexities associated with diagnosing viral co-infections, particularly in non-endemic areas. They described the case of a young Filipino woman presenting with symptoms consistent with mpox and varicella zoster virus (VZV). Despite inconclusive results from specific diagnostic tests, a high VZV viral load was confirmed, although mpox detection remains uncertain. This instance emphasizes the significance of differential diagnosis and the imperative to implement accurate diagnostic criteria viral co-infections. This paper advocates for heightened awareness and diagnostic precision, especially in non-endemic areas where healthcare professionals may lack familiarity with mpox manifestations.

Still further research is needed with global, regional, and national collaborative support to understand the scientific gaps to combat the current mpox outbreak. This will be achieved by implementing comprehensive surveillance and response strategies, advancing research, ensuring equitable access to medical countermeasures, minimizing zoonotic transmission, and empowering communities to actively participate in outbreak prevention and control (Mpox global strategic preparedness and response plan, 2024).
